# Sustained efficacy and safety of a 300IR daily dose of a sublingual solution of birch pollen allergen extract in adults with allergic rhinoconjunctivitis: results of a double-blind, placebo-controlled study

**DOI:** 10.1186/2045-7022-4-7

**Published:** 2014-02-11

**Authors:** Margitta Worm, Sabina Rak, Frédéric de Blay, Hans-Jorgen Malling, Michel Melac, Véronique Cadic, Robert K Zeldin

**Affiliations:** 1 Allergy-Centre-Charité, Charité - Universitätsmedizin Berlin, 10117 Berlin, Germany; 2 Asthma and Allergy Research Group, Department of Respiratory Medicine and Allergology, Sahlgrenska University Hospital, Göteborg, Sweden; 3 Chest Diseases Department, Strasbourg University Hospital, Federation of Translational Medicine, University of Strasbourg, Strasbourg, France; 4 Allergy Clinic, Gentofte University Hospital, Copenhagen, Denmark; 5 Stallergenes S.A., Antony, France

**Keywords:** Birch pollen, Tree pollen, Sublingual immunotherapy, Double-blind placebo-controlled trial, Allergic rhinoconjunctivitis, Allergen immunotherapy, Specific immunotherapy, Oral allergy syndrome, Sustained efficacy, Rhinoconjunctivitis Quality of Life Questionnaire

## Abstract

**Background:**

Allergic rhinoconjunctivitis (ARC) due to birch pollen is a growing health concern in Europe. Here, we report the efficacy and safety of 300IR birch pollen sublingual solution administered discontinuously for 2 consecutive years to patients with birch-associated allergic rhinoconjunctivitis.

**Methods:**

Birch pollen-allergic adults were randomized in this double blind study to 300IR birch pollen sublingual solution or placebo, daily, starting 4 months before and continuing through the pollen season for two pollen seasons. Randomization was stratified according to the presence or absence of oral allergy syndrome (OAS). The primary efficacy endpoint was the Average Adjusted Symptom Score (AAdSS) over the second pollen season and was analyzed by ANCOVA. Secondary efficacy endpoints included the AAdSS over the first pollen period. Safety was evaluated by means of adverse event monitoring.

**Results:**

574 patients (284 in the active group and 290 in the placebo group) were randomized and 496 completed the study. Over the second pollen period, the least square (LS) mean AAdSS was significantly lower in the 300IR group than in the placebo group (LS mean difference −2.04, 95% CI [−2.69, −1.40], (p <0.0001) with a relative reduction of 30.6%. Results were consistent in patients with and without OAS (−33.6% and −28.4%, respectively). A significant reduction in LS mean AAdSS was also observed over the first pollen season. The most frequently reported adverse events were application site reactions: oral pruritus, throat irritation, and mouth edema. There were no reports of anaphylaxis.

**Conclusions:**

Pre- and co-seasonal treatment with 300IR birch pollen sublingual solution demonstrated sustained clinical efficacy over 2 pollen seasons and was well tolerated in adults with birch pollen-associated allergic rhinoconjunctivitis. Efficacy results were consistent in patients with and without oral allergy syndrome.

**Trial registration:**

ClinicalTrials.gov: NCT01731249.

## Background

Birch is common in Northern Europe and is considered the major pollen-allergen-producing tree in Northern Europe. The pollen is generally present from March to May, depending on the latitude, and for up to 8 weeks [1]. Sensitization to birch correlates with the distribution of the tree, with low prevalence in Southern Europe and high prevalence in Northern Europe [2]. Cross-reactivity to alder and hazel contributes to a prolonged tree pollen season in patients with birch pollen allergy [3]. In addition to allergic rhinoconjunctivitis (ARC) symptoms, 50% to 93% of birch pollen allergic patients develop an oral allergy syndrome (OAS), with oropharyngeal symptoms after eating certain foods, because of the cross-reactivity between the major birch pollen allergen, Bet v 1, and the food proteins [4-6].

ARC represents a considerable burden on public health because of its prevalence, its impact on quality of life and productivity, its economic costs, and its associated co-morbidities, including asthma [7-9]. Current treatment options for ARC are allergen avoidance, symptomatic pharmacotherapy, and allergen immunotherapy (AIT). Limiting exposure to outdoor triggers is quite difficult for practical reasons. Symptomatic treatment options include antihistamines, intranasal corticosteroids, and leukotriene modifiers. These provide temporary relief from allergy symptoms but are not effective in all patients and are not disease-modifying [7]. WAO Guidelines recommend AIT by subcutaneous (SCIT) or sublingual (SLIT) route as therapeutic options for patients whose symptoms are not adequately controlled by avoidance measures or medications, those experiencing unacceptable adverse effects of medications, or those who wish to reduce the long-term use of medications [10,11].

The positive benefit-risk ratio of SLIT and its ease of use are likely factors that have contributed to its substantially increased use in Europe [10,12]. However, as there have been few large, double-blind, controlled AIT trials for birch [12,13], this study was conducted to assess the sustained efficacy (over two pollen seasons) and safety of pre- and co-seasonal treatment with 300IR (Index of Reactivity) birch-pollen sublingual solution in adults with birch-pollen induced ARC. The study also explored the efficacy and safety of treatment with the birch-pollen sublingual solution in the subpopulations with and without oral allergy syndrome.

## Methods

### Trial design

This was a randomized, double-blind, placebo-controlled study conducted at 56 study centers in 11 European countries (ClinicalTrials.gov number: NCT01731249).

**Figure 1:**
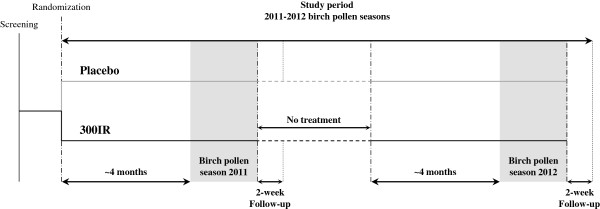
**Study design.** The no-treatment period was about 7 months.

Patients were enrolled between November 2010 and January 2011 for a two-year study consisting of two pre- and co-seasonal treatment periods in 2011 and 2012 (Figure 1).

Eligible patients were randomized 1:1 by center (block size of 4) using a computer-generated list to receive placebo or 300IR sublingual solution of birch pollen allergen extract initiated approximately 4 months before the expected start of the birch pollen season and continued throughout the season. Randomization was stratified according to the presence or absence of OAS and with the objective of enrolling a similar number of patients with and without OAS. Patients, investigators and all other study personnel remained blinded for the entire study. The study complied with ICH good clinical practice and was approved by local Regulatory Authorities and Independent Ethics Committees. Patients gave their written informed consent before performance of any study-related procedure. For a list of ethics committees which provided approval for this study, please see Additional file 1.

### Patients

The study enrolled men and women aged 18 to 65 with documented birch pollen-related ARC for at least the two previous birch pollen seasons that required intake of symptomatic treatments. Sensitivity to birch pollen was verified by a positive Skin Prick Test (Stallergenes S.A.), defined as wheal diameter > 3 mm, birch-specific serum IgE (ImmunoCAP, Thermoscientific) ≥ 0.70 kUA/L, and a Retrospective Rhinoconjunctivitis Total Symptom Score (RRTSS) ≥ 12 (0–18 scale) based on the most severe days during one of the two birch pollen seasons preceding enrolment. Patients were excluded from the study if they had symptoms of rhinitis/rhinoconjunctivitis during the birch pollen season due to any other allergen (except alder and hazel), had received AIT treatment to birch pollen and/or another Betulaceae species (such as hazel or alder) within the previous 5 years, were receiving AIT for any other allergen, or had moderate or severe persistent asthma. Patients with asthma controlled with inhaled corticosteroids at a maximum dose of 400 μg of budesonide or the equivalent were eligible.

### Study treatment and rescue medications

Active treatment consisted of a sublingual solution of birch-pollen allergen standardized extract (Staloral, Stallergenes S.A., Antony, France) provided at a concentration of 10IR/mL for the titration phase and 300IR/mL for both the titration and maintenance phases. IR is the in-house standardization unit used to quantify the allergenicity of a solution. The titer of an allergen extract solution corresponds to 100IR/mL when, in an SPT performed with the Stallergenes’ SPT device (Stallerpoint®), in 30 subjects sensitized to the allergen in question, the extract produces a wheal measuring 7 mm in diameter (geometric mean).

Each year, treatment was initiated with a 12-day titration phase during which the dose was progressively increased daily from 1IR to 300IR. The dose regimen was as follows: 1, 2, 4, 6, 8, 10, 30, 60, 120, 180, 240, and 300IR/day. The maintenance phase consisted of daily intake of a 300IR dose until the end of the birch pollen season. Patients were instructed to deposit the prescribed dose directly under the tongue and keep it there for 2 minutes before swallowing. Each year, the first dose of treatment was taken at the study site and the patients were monitored for 30 minutes; the remainder of the treatment was taken at home. To maintain blinding, the placebo sublingual solution was matched in appearance, color and taste (i.e., the same glycerol saline solution was used and a coloring agent added for color matching).

Rescue medications (oral and ophthalmic antihistamines and nasal corticosteroids) were provided to patients to be used according to a stepwise regimen to manage severe ARC symptoms due to birch pollen. The investigator could provide oral corticosteroids if other rescue medications were insufficient.

### Assessments

At the beginning of each treatment period, patients received an electronic diary (PHT Corporation, Geneva, Switzerland) and were advised to record, on a daily basis, their intake of the investigational product, the severity of each of their six rhinoconjunctivitis symptoms (sneezing, rhinorrhea, nasal pruritus, nasal congestion, ocular pruritus and watery eyes) on a 4-point descriptor scale from 0 (absent) to 3 (severe) and their use of rescue medication during the previous 24 hours.

Each study year, the patients completed the standardized Rhinoconjunctivitis Quality of Life Questionnaire (RQLQ[S])[14] before the first intake of the investigational product (i.e., 4 months before the expected start of the birch pollen period), then every week from 2 weeks before the expected start of the birch pollen season to 2 weeks after its end (i.e., end of the treatment period).

Birch-specific serum IgE and IgG4 were measured using fluoroenzyme immunoassay at study entry, about 2 weeks after the end of the first pollen season, about 4 months before the second pollen period and about 2 weeks after the end of the second pollen season (ImmunoCAP, Thermoscientific).

Safety variables were adverse events (AEs) monitored from the signing of patient informed consent as well as data from physical examinations and clinical laboratory assessments. For each treatment period, treatment-emergent adverse events (TEAEs) were defined as adverse events which started on or after the first administration of the investigational product, up to 30 days after its last intake.

### Outcomes

The sum of the six Rhinoconjunctivitis Symptom Scores (RSS) defined the daily Rhinoconjunctivitis Total Symptom Score (RTSS, range 0–18). The daily Rescue Medication Score (RMS) was derived as follows: 0 = no rescue medication taken; 1 = use of antihistamines (oral and/or ophthalmic); 2 = use of nasal corticosteroids; 3 = use of oral corticosteroids. If more than one class of rescue medication was taken on a particular day, the highest score was retained for the score of that day.

The primary efficacy variable was the Average Adjusted Symptom Score (AAdSS, range 0–18) during the second birch pollen period. The AAdSS takes into account both symptom scores and rescue medication usage. It is subject specific and is calculated as the average of the daily Adjusted Symptom Scores (AdSSs) over the evaluation period. Each day, the AdSS adjusts the daily RTSS for rescue medication usage. If a patient took rescue medication on one day, the AdSS of that day is defined as the RTSS of that day or the AdSS of the day before, whichever was higher. The next day, the AdSS is defined as the RTSS of that day or the AdSS of the day before, whichever was higher [15]. Secondary efficacy variables included the Average RTSS (ARTSS), individual Average RSSs (ARSSs), Average RMS (ARMS), and overall RQLQ(S) score.

### Birch pollen periods

The estimated site-specific start and end dates of the 2011 and 2012 birch pollen seasons were supplied by SciCon Pharma Sciences (Vienna, Austria) taking into account historical pollen data and seasons for the various sites. Actual daily pollen counts and pollen graphs of the 2011 and 2012 birch pollen seasons were provided so that the start and end of the pollen seasons at each site were defined before data unblinding. The birch-pollen period at each site was defined as the period covering 90% of the total annual birch-pollen count.

### Statistical analyses

Based on previous birch pollen studies, given an α = 0.05 and a coefficient of variation of the primary endpoint of 70% (CV = 0.70), a sample size of 210 evaluable patients per treatment group would provide 90% power to detect at least a 20% relative mean difference vs. placebo, which is the threshold recommended by the WAO taskforce as clinically relevant for efficacy [16,17]. Assuming a dropout rate of 12% per year, it was planned to randomize 544 patients (i.e., 272 per treatment group).

Statistical analyses were performed using the SAS System® for Windows version 9.1 or higher (SAS Institute, Cary, North Carolina, USA). The threshold for statistical significance was set at p < 0.05 and all inferential tests were 2-sided.

The Full Analysis Sets for Period 1 (FAS_P1_) and Period 2 (FAS_P2_) included all patients who received at least one dose of the investigational product during that period and had at least one AdSS during the corresponding pollen period.

The primary endpoint (i.e., the AAdSS during the second birch pollen period for the FAS_P2_) was analyzed using an analysis of covariance (ANCOVA) with treatment as main effect, OAS status and pooled centre as stratification variables, and age in categories, gender, asthma status and baseline sensitization status as covariates. The primary efficacy analysis was repeated on the subgroups of patients with and without OAS. ARTSS, ARMS, and ARSSs were analyzed as per the primary efficacy criterion. The RQLQ(S) was analyzed using a linear mixed effects model with the weekly overall RQLQ(S) scores during the birch pollen period as dependent variables and treatment group, week number, baseline RQLQ(S) overall score, pooled centre, OAS status, age, gender, asthma and sensitization status as independent variables. The same analyses were repeated for the first pollen period (FAS_P1_).

## Results

**Figure 2:**
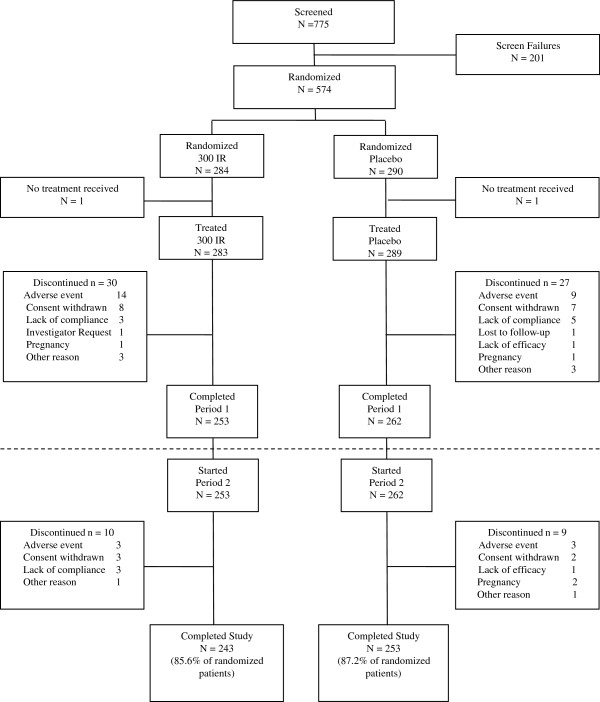
**Patient disposition.** Patients were randomized to receive either placebo or 300IR sublingual solution of birch allergen extract initiated approximately 4 months before the birch pollen season and continued through it. Patients were treated discontinuously for two consecutive years.

A total of 574 patients were randomized and 572 received at least one dose of the investigational product: 283 in the 300IR group and 289 in the placebo group. Of these, 253 patients in the 300IR group and 262 patients in the placebo group completed the first period and started the second period of treatment. A total of 496 patients completed the study (Figure 2).

Demographics and baseline characteristics were similar in the two groups at study entry and remained so at the start of the second study year (Table 1). At study entry, the average patient had a history of birch-associated allergic rhinitis for about 14 years, more than 68% were polysensitized, 20% had asthma, and about 54% had OAS.

**Table 1:** **Demographics and baseline characteristics (FAS**_**P1**_**, FAS**_**P2**_**)**

**PERIOD 1**		
	**Placebo**	**300IR**
	**N = 261**	**N = 275**
Age (years)	36.6 (11.26)	38.6 (10.97)
Gender (n and % female)	137 (52.5)	141 (51.3)
Duration of allergic rhinoconjunctivitis (years)	13.7 (10.72)	14.2 (10.52)
Presence of Oral Allergy Syndrome (n, %)	141 (54.0)	150 (54.5)
FEV_1_ (% predicted)	100.7 (11.82)	101.8 (13.80)
RRTSS	14.2 (1.62)	14.4 (1.75)
Presence of asthma (n, %)	75 (28.7)	58 (21.1)
Poly-sensitized^*^ (n, %)	179 (68.6)	205 (74.5)
**PERIOD 2**		
	**Placebo**	**300IR**
	**N = 247**	**N = 253**
Age (years)	36.7 (11.33)	38.4 (11.05)
Gender (n and % female)	129 (52.2)	132 (52.2)
Duration of allergic rhinoconjunctivitis (years)	13.4 (10.39)	13.8 (10.49)
Presence of Oral Allergy Syndrome (n, %)	133 (53.8)	138 (54.5)
FEV_1_ (% predicted)	100.8 (12.05)	102.1 (13.58)
RRTSS	14.2 (1.60)	14.4 (1.75)
Presence of asthma (n, %)	70 (28.3)	54 (21.3)
Poly-sensitized^*^ (n, %)	169 (68.4)	185 (73.1)

Results describing continuous variables are expressed as means (SDs). Results describing categorical variables are expressed as the number of patients and the percentage relative to the number of patients in the FAS_P1_ and FAS_P2_ with non-missing data.

RRTSS = Retrospective Rhinoconjunctivitis Total Symptom Score.

^*^Sensitized to birch pollen and at least one other allergen tested.

As treatment was initiated 4 months before the pollen season and continued for its duration, there could be some variation in treatment duration linked to the length of the pollen period at each site. Overall, patients were treated for approximately 5 months in each of the 2 years of the study. The mean treatment duration was 146 days for the 300IR group and 145 days for the placebo group in Period 1, and 168 days and 169 days, respectively in Period 2.

### Efficacy outcomes

**Figure 3:**
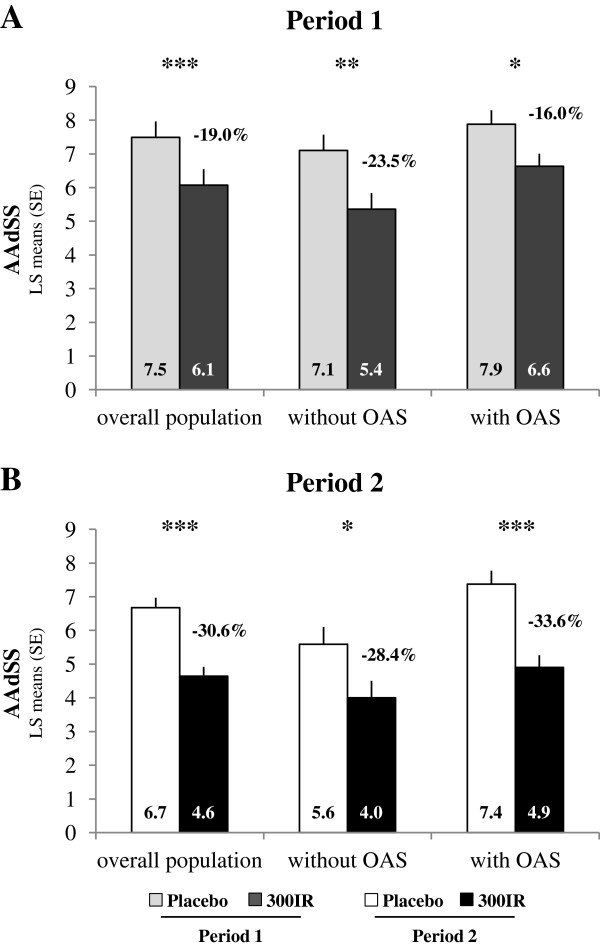
**Average Adjusted Symptom Score.** Data are presented for the overall population and by baseline OAS status during Periods 1 **(A)** and 2 **(B**, Primary Endpoint) in the FAS_P1_ and FAS_P2_. ANCOVA *p < 0.05; **p < 0.001; ***p < 0.0001.

During the second birch pollen period, the AAdSS, which adjusts symptom scores for the use of rescue medication, was significantly lower in the 300IR group compared to placebo (Figure 3B) with a difference in LS means of −2.04 (95% CI [−2.69; −1.40], p < 0.0001), corresponding to a relative LS means difference of −30.6%. Over the first pollen period, the difference was −1.42 (95% CI [−2.07; −0.77], p < 0.0001). This corresponds to a relative LS means difference of −19.0%.

The same analyses performed in the subgroups of patients with and without OAS showed statistically significant differences in AAdSS compared to placebo for both periods (Figure 3A, B). Relative LS means differences of −28.4% in patients without OAS and −33.6% in patients with OAS were observed during the second pollen period.

**Figure 4:**
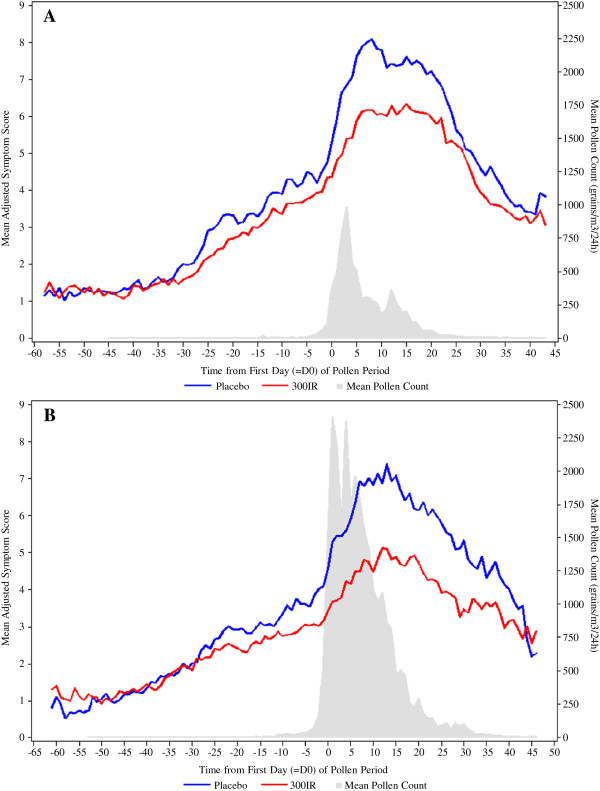
**Daily mean Adjusted Symptom Score and birch pollen season.** Data are presented for Periods 1 **(A)** and 2 **(B)** in the FAS_P1_ and FAS_P2_. The daily mean birch pollen count (grains/m^3^) was the average of pollen count weighted by the number of patients within the catchment area of each pollen trap.

Throughout each pollen period, the mean daily AdSSs increased with the pollen exposure at each catchment area and the daily AdSSs of the placebo group were always higher than those of the 300IR group (Figure 4).

**Figure 5:**
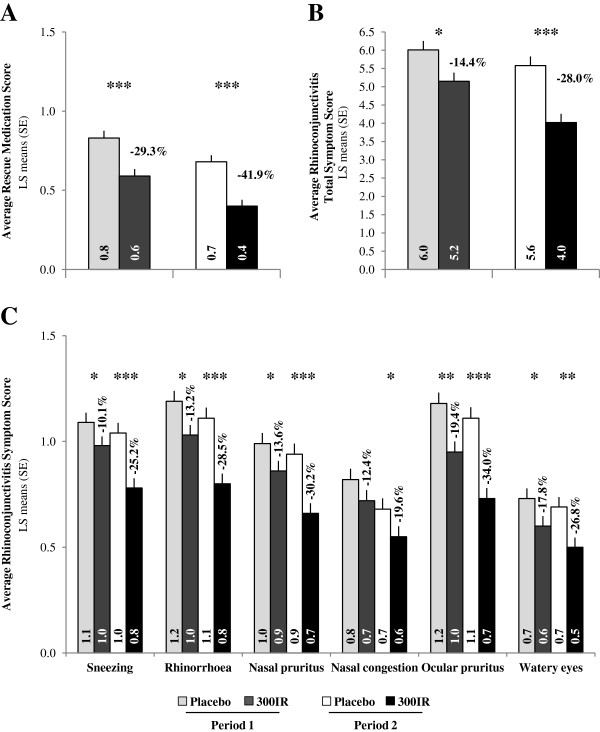
**Symptoms and medication scores.** Average Rescue Medication Score **(A)**, Average Rhinoconjunctivitis Total Symptom Score **(B)** and Average Rhinoconjunctivitis Symptom Scores **(C)** for Periods 1 and 2 in the FAS_P1_ and FAS_P2_. ANCOVA *p < 0.05; **p < 0.001; ***p < 0.0001.

During both pollen periods, the use of rescue medication as reflected by the ARMS was significantly reduced in the 300IR group compared to the placebo group (p < 0.0001), with relative LS mean differences of −29.3% and −41.9%, respectively (Figure 5A). During the second pollen period, this resulted in a significant reduction (p < 0.0001) in the proportion of patients who used at least one rescue medication in the 300IR group (60.7%) compared to the placebo group (81.8%). For each class of rescue medication, the proportion of days rescue medications were used was higher in the placebo group.

When considering symptoms alone, there was a statistically significant difference in ARTSS compared to placebo during the second pollen period (p < 0.0001), with a corresponding LS means difference of −28.0%. Similar results were observed during the first pollen period (Figure 5B). Each of the six ARSSs were lower in the 300IR group compared to placebo during both pollen periods, with corresponding relative LS means differences ranging from −10.1% to −19.4% during the first pollen period and from −19.6% to −34.0% during the second pollen period (Figure 5C), and statistically significant differences for all symptom scores during the second pollen period. For both pollen periods, the largest reduction in symptom severity in the active treatment group compared to placebo was observed for ocular pruritus.

**Figure 6:**
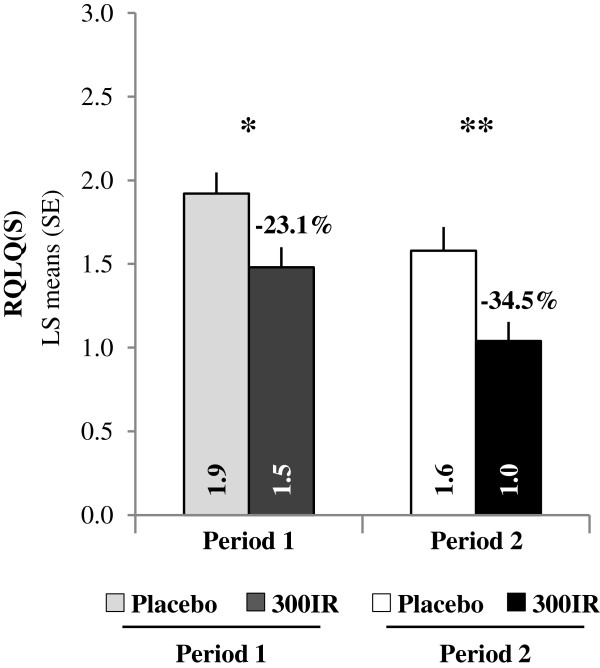
**Standardized Rhinoconjunctivitis Quality of Life Questionnaire (RQLQ[S]) overall scores.** Data are presented for birch pollen Periods 1 and 2 in the FAS_P1_ and FAS_P2_. Linear mixed effects model with repeated measures *p < 0.05; **p < 0.001.

Each pollen period, a significant difference between the 300IR and placebo groups was shown for the overall RQLQ(S) scores. During the first pollen period, the overall RQLQ(S) LS mean difference was −0.44 (95% CI [−0.74; −0.15]) compared to placebo, corresponding to a relative LS means difference of −23.1%. During pollen period 2, the RQLQ(S) LS mean difference was of −0.55 (95% CI [−0.85; −0.24]) compared to placebo, corresponding to a relative LS means difference of −34.5% (Figure 6).

### Serum immunological markers

**Figure 7:**
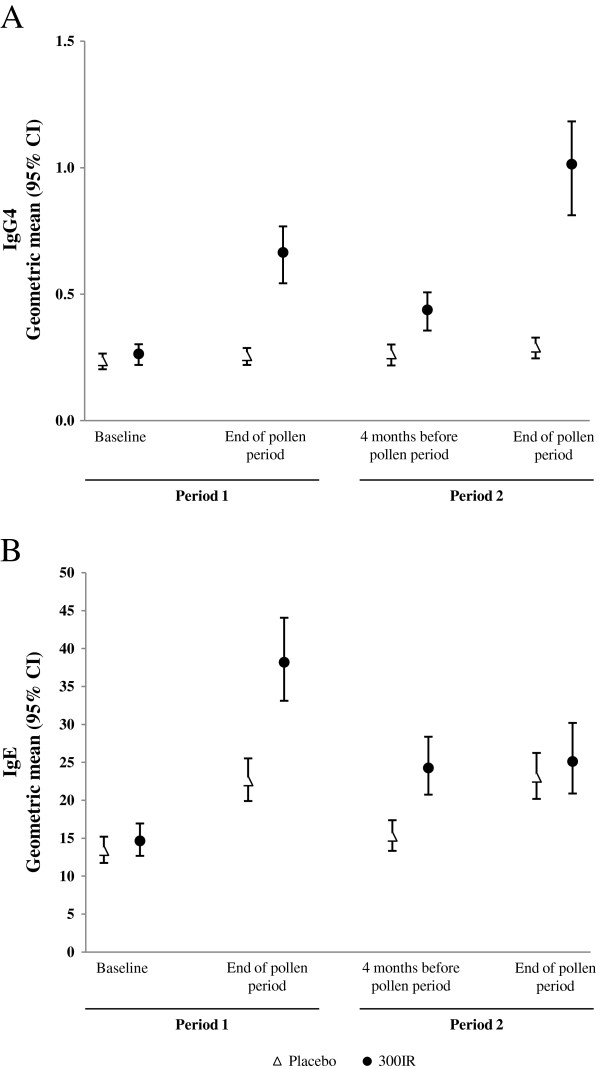
**Immunological markers (FAS**_**P1**_**, FAS**_**P2**_**).** Serum specific-IgG4 levels **(A)** and serum specific-IgE levels **(B)** at baseline, at the end of pollen period (Period 1), 4 months before the pollen period (Period 2) and at the end of the pollen period (Period 2).

At study entry, birch-specific serum IgE and IgG_4_ were similar in the 300IR and placebo groups. At the end of the second pollen period, the change from baseline in birch-specific serum IgE was similar in the two treatment groups, however, birch-specific serum IgG_4_ increased by about four-fold in the 300IR group but was essentially unchanged in the placebo group (Figure 7).

### Safety

For each study period, the safety set included all patients who received at least one dose of the investigational product: 572 patients in Period 1 and 511 in Period 2.

There were no deaths during the study and no reports of anaphylaxis. Ten patients reported serious TEAEs during Period 1: 8 in the active group (pneumonia, erysipelas, abscess of external auditory meatus, syncope, colitis, urticaria, hemorrhagic stroke, and pancreatitis) and 2 in the placebo group (bronchopneumonia and pulmonary malformation). Among them, urticaria was the only event considered related to treatment by the investigator. During the second Period, 5 patients reported serious TEAEs: 2 in the active group (sciatica and metastatic colon cancer) and 3 in the placebo group (hemiparesis, peritoneal adhesions and subarachnoid hemorrhage); none were considered drug-related.

A similar percentage of patients reported TEAEs in the 2 groups: 70.7% in the active group and 64.0% in the placebo group. The most frequent TEAEs were application site reactions: oral pruritus, mouth edema and throat irritation (Table 2). During Period 2, 46.8% in the active group and 48.6% in the placebo group reported TEAEs, the most frequent being oral pruritus. As sublingual immunotherapy entails direct exposure of the oral mucosa to the allergen responsible for the OAS, the safety profile was also considered in the subgroups of patients with and without OAS. During Period 1, the incidence of TEAEs in patients with OAS was similar in the two treatment groups (78.3% in the active group and 73.4% in the placebo group) and was higher than in patients without OAS (61.8% in the active group and 52.7% in the placebo group). This was also observed during Period 2.

**Table 2:** Incidence of TEAEs reported by at least 5% of participants in either treatment group

**System organ class** Preferred term	**Overall population**		**With OAS**		**Without OAS**	
	**300IR**	**Placebo**	**300IR**	**Placebo**	**300IR**	**Placebo**
	**(N = 283)**	**(N = 289)**	**(N = 152)**	**(N = 158)**	**(N = 131)**	**(N = 131)**
	**n (%)**	**n (%)**	**n (%)**	**n (%)**	**n (%)**	**n (%)**
**PERIOD 1**						
Patients who had at least one TEAE	200 (70.7)	185 (64.0)	119 (78.3)	116 (73.4)	81 (61.8)	69 (52.7)
**Gastrointestinal disorders**	**126 (44.5)**	**54 (18.7)**	**83 (54.6)**	**36 (22.8)**	**43 (32.8)**	**18 (13.7)**
Oral pruritus	70 (24.7)	11 (3.8)	47 (30.9)	7 (4.4)	23 (17.6)	4 (3.1)
Edema mouth	28 (9.9)	0 (0.0)	21 (13.8)	0 (0.0)	7 (5.3)	0 (0.0)
**Infections and infestations**	**89 (31.4)**	**106 (36.7)**	**57 (37.5)**	**71 (44.9)**	**32 (24.4)**	**35 (26.7)**
Nasopharyngitis	31 (11.0)	43 (14.9)	23 (15.1)	26 (16.5)	8 (6.1)	17 (13.0)
**Respiratory, thoracic, mediastinal disorders**	**64 (22.6)**	**43 (14.9)**	**42 (27.6)**	**30 (19.0)**	**22 (16.8)**	**13 (9.9)**
Throat irritation	19 (6.7)	2 (0.7)	14 (9.2)	2 (1.3)	5 (3.8)	0 (0.0)
**Nervous system disorders**	**53 (18.7)**	**53 (18.3)**	**38 (25.0)**	**37 (23.4)**	**15 (11.5)**	**16 (12.2)**
Headache	49 (17.3)	44 (15.2)	34 (22.4)	31 (19.6)	15 (11.5)	13 (9.9)
**Musculoskeletal and connective tissue disorders**	**25 (8.8)**	**27 (9.3)**	**17 (11.2)**	**15 (9.5)**	**8 (6.1)**	**12 (9.2)**
**Eye disorders**	**22 (7.8)**	**27 (9.3)**	**17 (11.2)**	**17 (10.8)**	**5 (3.8)**	**10 (7.6)**
**Skin and subcutaneous tissue disorders**	**13 (4.6)**	**17 (5.9)**	**7 (4.6)**	**15 (9.5)**	**6 (4.6)**	**2 (1.5)**
**PERIOD 2**						
	**300IR**	**Placebo**	**300IR**	**Placebo**	**300IR**	**Placebo**
	**(N = 252)**	**(N = 259)**	**(N = 134)**	**(N = 142)**	**(N = 118)**	**(N = 117)**
	**n (%)**	**n (%)**	**n (%)**	**n (%)**	**n (%)**	**n (%)**
Patients who had TEAE	118 (46.8)	126 (48.6)	68 (50.7)	80 (56.3)	50 (42.2)	46 (39.3)
**Infections and infestations**	**65 (25.8)**	**77 (29.7)**	**41 (30.6)**	**50 (35.2)**	**24 (20.3)**	**27 (23.1)**
Nasopharyngitis	19 (7.5)	34 (13.1)	12 (9.0)	22 (15.5)	7 (5.9)	12 (10.3)
**Gastrointestinal disorders**	**41 (16.3)**	**23 (8.9)**	**27 (20.1)**	**15 (10.6)**	**14 (11.9)**	**8 (6.8)**
Oral pruritus	21 (8.3)	2 (0.8)	14 (10.4)	2 (1.4)	7 (5.9)	0 (0)
**Nervous system disorders**	**17 (6.7)**	**21 (8.1)**	**11 (8.2)**	**19 (13.4)**	**6 (5.1)**	**2 (1.7)**
Headache	13 (5.2)	17 (6.6)	10 (7.5)	16 (11.3)	3 (2.5)	1 (0.9)
**Respiratory, thoracic, mediastinal disorders**	**14 (5.6)**	**21 (8.1)**	**10 (7.5)**	**14 (9.9)**	**4 (3.4)**	**7 (6.0)**
**Musculoskeletal and connective tissue disorders**	**14 (5.6)**	**18 (6.9)**	**7 (5.2)**	**12 (8.5)**	**7 (5.9)**	**6 (5.1)**

Safety Set_P1_, Safety Set_P2_. Adverse events were classified according to their system organ class and preferred term (MedDRA version 14.0).

TEAEs were adverse events occurring during the treatment period up to 30 days after the last treatment administration.

n = number of patients with at least one event in given preferred term; % = percentage of patients with at least one event relative to the number of patients in each treatment group (N) in the Safety Sets.

A total of 22 patients withdrew due to TEAEs during Period 1: 13 in the active group (including 9 with OAS) and 9 in the placebo group (including 5 with OAS). Among these, the most common TEAEs were local reactions and were considered related to the treatment. During the second treatment period, 5 patients (3 in the active group and 2 in the placebo group) withdrew due to TEAEs.

## Discussion

Earlier birch pollen AIT studies have shown positive outcomes [18-21], but have either included small numbers of patients [18-20] or were observational [21,22]. The need for larger scale studies of sublingual immunotherapy has long been acknowledged [17].

The present study is the largest to date specifically focused on assessing the efficacy and safety of a sublingual solution of birch pollen allergen extract as treatment for allergic rhinitis. After two consecutive pre- and co-seasonal treatment periods, a significant, sustained reduction in symptoms and medication use, measured with the Average Adjusted Symptom Score, was demonstrated for the 300IR dose. The robustness of this finding was supported by the positive results on secondary endpoints including efficacy during the first birch pollen period.

Evaluation of treatment efficacy included the assessment of patient self-reported quality of life. Each pollen period, a significant difference in RQLQ(S) overall score was observed between active treatment and placebo. For the second pollen period, the adjusted mean difference vs. placebo was greater than 0.5, the intra-patient minimal clinically important difference defined for symptomatic treatments [23]. Of note, in this study rescue medication was permitted as treatment for severe rhinoconjunctivitis symptoms and was used more frequently in the placebo group. Therefore, the true impact of treatment with the sublingual solution of birch pollen allergen extract on patients’ quality of life as evaluated by the RQLQ was underestimated.

Randomization was stratified on the presence or absence of OAS. Efficacy of the birch-pollen sublingual solution was similar in the subgroups with and without OAS, with statistically significant reductions in overall symptom scores (i.e., AAdSS) and in the use of rescue medication, compared to placebo, regardless of OAS status.

As shown in Figure 4, the pollen count was notably higher during the second pollen season, (peak nearly 2400 grains/m^3^/24 h), compared to the first pollen season (peak at about 1000 grains/m^3^/24 h). In spite of the higher pollen exposure, the relative adjusted mean difference in AAdSS in the active group relative to the placebo group was higher during the second pollen period compared to the first pollen period.

In this study, birch pollen allergen extract sublingual solution was associated with an acceptable safety profile. There were no reports of anaphylaxis or serious severe laryngopharyngeal reactions. The most frequent adverse events were application site reactions (e.g., oral pruritus and throat irritation). Consistent with the published experience with grass pollen sublingual immunotherapy, the incidence of withdrawals due to adverse events decreased from the first treatment period to the second [24,25].

The sustained clinical efficacy over 2 years, favorable safety profile, and ability to administer birch pollen sublingual immunotherapy at home, make this treatment an alternative to subcutaneous immunotherapy for patients with birch pollen-associated ARC.

## Conclusions

In this European study, 4 months of pre-seasonal and co-seasonal treatment with 300IR sublingual solution of birch-pollen extract over 2 consecutive years demonstrated sustained and clinically meaningful efficacy and acceptable safety profile in adults with birch pollen-associated allergic rhinoconjunctivitis. Efficacy and safety were shown in the subpopulations of patients with and without oral allergy syndrome.

## Abbreviations

AAdSS: Average Adjusted Symptom Score; AdSS: Adjusted Symptom Score; AE: Adverse event; AIT: Allergen immunotherapy; ANCOVA: Analysis of covariance; ARC: Allergic rhinoconjunctivitis; ARMS: Average Rescue Medication Score; ARTSS: Average Rhinoconjunctivitis Total Symptom Score; ARSS: Average Rhinoconjunctivitis Symptom Score; CI: Confidence interval; FAS: Full Analysis Set; IR: Index of reactivity; LS: Least square; OAS: Oral allergy syndrome; RMS: Rescue Medication Score; RRTSS: Retrospective Rhinoconjunctivitis Total Symptom Score; RQLQ(S): Standardized Rhinoconjunctivitis Quality of Life Questionnaire; RTSS: Rhinoconjunctivitis Total Symptom Score; RSS: Rhinoconjunctivitis Symptom Score; SCIT: Subcutaneous immunotherapy; SE: Standard error; SLIT: Sublingual immunotherapy; TEAE: Treatment-emergent adverse event.

## Competing interests

MW was the principal investigator of the study and she also received honoraria for lectures from Stallergenes. SR received research grants, been speaker and served on advisory board for Stallergenes and also participated in clinical studies. She has been a speaker and received grants from Allergopharma and speaker for ALK. SR served on advisory board for Circassia and received research grants from Allergis. FdB has received grants and personal fees from Stallergènes, ALK, Novartis, and Mundipharma. HJM has received research grants, been a speaker and served on advisory boards for Stallergenes, Allergopharma, ALK-Abelló, Biomar and Novartis. MM, VC, RKZ are employees of Stallergenes S.A.

## Authors’ contributions

MW was involved in the design of the study and the leading principal investigator. She assessed the data of the study and supported intellectually the writing of the manuscript and revised the article. SR 1) contributed with and conducted study with a large number of patients; 2) has been involved in revising the manuscript critically for important intellectual content; and 3) have given final approval of the version to be published; and 4) agrees to be accountable for all aspects of the work in ensuring that questions related to the accuracy or integrity of any part of the work are appropriately investigated and resolved. FdB contributed to conception and design of the study, acquisition, analysis and interpretation of data and revised the manuscript critically for important intellectual content. HJM: 1) has made substantial contributions to conception and design, acquisition of data, analysis and interpretation of data; 2) has been involved in revising the manuscript critically for important intellectual content; and 3) have given final approval of the version to be published; 4) agrees to be accountable for all aspects of the work in ensuring that questions related to the accuracy or integrity of any part of the work are appropriately investigated and resolved. MM was involved in the conception and design of the study, analysis and interpretation of data and revised the manuscript critically for important intellectual content. VC contributed to the statistical analysis of the data and their interpretation and revised the manuscript critically for important intellectual content. RKZ supervised the clinical team, contributed to the analysis and interpretation of data and revised the manuscript critically for important intellectual content. All authors read and approved the final manuscript.

## Supplementary Material

Additional file 1 List of ethics committees. Click here for file
