# Supplementary material for: Sustained efficacy and safety of a 300IR daily dose of a sublingual solution of birch pollen allergen extract in adults with allergic rhinoconjunctivitis: results of a double-blind, placebo-controlled study
Source: Clin Transl Allergy. 2014 Feb 11;4:7. doi: 10.1186/2045-7022-4-7 (PMC3928083; doi:10.1186/2045-7022-4-7)
Supplement: Additional file 1 — List of ethics committees. [file 2045-7022-4-7-S1.docx]

**List of Central Ethics Committees which approved the study**

**CZECH REPUBLIC**

Eticka komise pro multicentricke klinicke hodnoceni FN Motol

V uvalu 84

Prague 5 150 06 - Czech Republic

**DENMARK**

Den Videnskabsetiske Komité for Region Midtlylland

Sundhedssekretariatet

Skottenborg 26, postboks 21

Viborg 8800 - Denmark

**ESTONIA**

ERC on Human Research of the University of Tartu

Lossi 3

Tartu 51003 - Estonia

**FINLAND**

HUS Medisiininen eettinen toimikunta

Biomedicum Helsinki "C, 7.krs

Tukholmankatu 8C

Helsinki 00029 HUS - Finland

**FRANCE**

CPP EST IV

1 place de l'Hôpital

Strasbourg 67091 - France

**GERMANY**

EC Berlin

Fehrbelliner Platz 1

Berlin 10707 - Germany

**LATVIA**

Ethics Committee For Clinical Trials On Medicinal Products

Aizkraukles street 21 - 113

Riga LV1006 - Latvia

**LITHUANIA**

Lithuanian Bioethics Committee

Didzioji g. 22

Vilnius LT-01128 - Lithuania

POLAND

Komisja Bioetyki UM w Łodzi

Al. Kosciuszki 4

Lodz 90-419 - Poland

**SLOVAKIA**

EC - FNsP F.D. Roosevelta

Nam. L. Svobodu 1

Banska Bystrica 97517 - Slovakia

**SWEDEN**

Regionala Etikprövningsnämnden i Göteborg Guldhedsgatan 5A

Box 401

Göteborg 40530 - Sweden
